# Preparation and Analysis of Two-Dimensional Four-Qubit Entangled States with Photon Polarization and Spatial Path

**DOI:** 10.3390/e24101388

**Published:** 2022-09-29

**Authors:** Jiaqiang Zhao, Meijiao Wang, Bing Sun, Lianzhen Cao, Yang Yang, Xia Liu, Qinwei Zhang, Huaixin Lu, Kellie Ann Driscoll

**Affiliations:** School of Physics and Electronic Information, Weifang University, Weifang 261061, China

**Keywords:** quantum information, entanglement, spontaneous down conversion, quantum state tomography, entanglement witness, Ardehali inequality

## Abstract

Entanglement states serve as the central resource for a number of important applications in quantum information science, including quantum key distribution, quantum precision measurement, and quantum computing. In pursuit of more promising applications, efforts have been made to generate entangled states with more qubits. However, the efficient creation of a high-fidelity multiparticle entanglement remains an outstanding challenge due to the difficulty that increases exponentially with the number of particles. We design an interferometer that is capable of coupling the polarization and spatial paths of photons and prepare 2-D four-qubit GHZ entanglement states. Using quantum state tomography, entanglement witness, and the violation of Ardehali inequality against local realism, the properties of the prepared 2-D four-qubit entangled state are analyzed. The experimental results show that the prepared four-photon system is an entangled state with high fidelity.

## 1. Introduction

Quantum entanglement is the basic resource in quantum information processing. In recent years, a variety of entanglement schemes have been proposed and verified, such as multiphoton schemes [1], cold atom schemes [2,3], quantum dot schemes [4], etc. The quantum information processing theory and experiments have been developed rapidly [5], especially the multiphoton entanglement, which plays an important role not only in the basic test of quantum nonlocality [6,7,8], but also in optical quantum computing [9,10], quantum teleportation [11,12] quantum key distribution (QKD) [13], and many other aspects. Over the past years, great efforts have been devoted to generating and manipulating more qubits. At the same time, many efforts are also being made to study the theoretical predictions of quantum mechanics based on polarization-entangled photons [14]. In experiments, the entangled photon systems are usually prepared by the spontaneous parametric down-conversion (SPDC) process in nonlinear crystal. In the conventional protocols for quantum information processing, the entanglement in one degree of freedom of photon systems is selected in the SPDC process. However, the difficulty of preparing a multiparticle entanglement increases exponentially with the number of particles because of the low multiphoton coincidence count rate and the high double-pair emission noise effect [15]. A single photon is able to carry more than just a qubit of quantum information, and when two photons are entangled in more than one degree of freedom, higher-dimensional (H-D) entanglement can be realized. In experiments, multidegree of freedom entanglement can be generated by the combination of the techniques used for creating entanglement in a single degree of freedom [16]. With this method, many different types of H-D entangled states can be prepared [17], such as the polarization–spatial H-D entangled state [18], polarization–orbital angular momentum H-D entangled state [19], etc. These H-D entangled systems have also been demonstrated experimentally. H-D entanglement is a fascinating resource for quantum communication and quantum computation. For example, it can improve the channel capacity of quantum communication and speed up quantum computation significantly.

On the other hand, people are also very interested in the nature and property description of entanglement. To date, people have found a variety of methods to describe the entanglement of the system. The fidelity measurement of the extent to which the desired state is created is the overlap of the experimentally produced state with the ideal one. The fidelity can be calculated using the density matrix combination with full state tomography [20]. The Einstein, Podolsky, and Rosen (EPR) paradox stimulated Bell to propose an inequality to detect the existence of entanglement in a two-particle system [21,22,23]. It has been proved that the violation of an inequality against local reality (LR) is a sufficient condition for the confirmation of entanglement. The entangled witnesses can also be used to detect the entanglement of the system. It can be obtained by measuring the Pauli operator of different angles [24,25].

Many methods have been devised to prepare multiparticle entangled states through H-D entangled states. These works provide a good entanglement source for the research and application of quantum information technology [16,17,18,19]. However, some systems are complex [26], and some require harsh experimental conditions such as low temperature [27]. In this paper, motivated by facilitating the generation of multiqubit entangled states, we have designed an interferometer that can couple the polarization and spatial paths of photons and prepare an entangled state of the two modes. As a basis, two photon entangled states with high brightness and high fidelity were prepared using SPDC technology. Next, we designed the composite interferometer and prepared two-dimensional (2-D) four-qubit Greenberger–Horne–Zeilinger (GHZ) entanglement states for the polarization and spatial path of photons. Finally, the properties of the prepared four-qubit entangled state were analyzed by three methods: quantum state tomography, entanglement witness, and the violation of Ardehali inequality against local realism (LR). The experimental results proved that the prepared 2-D four-photon system is an entangled state with high fidelity.

## 2. Experimental Preparation of a 2-D Four-Qubit Entangled State

**Figure 1:**
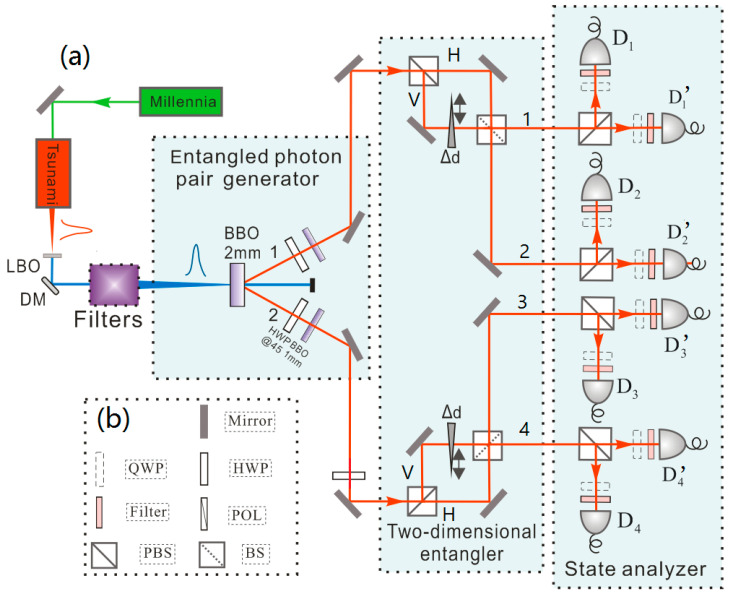
(Color online) (**a**) Scheme of the experimental setup. The average power of a UV laser behind filters in the experiment is 100 mW. Filters are formed by five DMs which can reflect a UV laser and transmit an IR laser. The 2-D entangler consists of PBS, BS, mirrors, and a quartz plate. The quartz plate Δd is used to adjust the phase φ between the photons *H* and V to ensure that the two photons reach the BS simultaneously. The coincidence time-window is set to be 5 ns, which ensures that accidental coincidence is negligible. Every output is spectrally filtered Δ_FWHM_ = 3 nm and monitored by fiber coupled single-photon detectors. The state analyzer is structured by PBS, QWP, a filter, and a single-photon detector (SPCM-AQRH-13-FC, integrated detection efficiency 60%). (**b**) Optical device description.

The first step in preparing a 2-D four-qubit entangled state in this experiment is the preparation of a two-photon entangled state with high brightness and fidelity. We produce the polarization-entangled two-photon entangled states using SPDC [28]. The experimental setup is shown in Figure 1. Using a CW 532 nm all-solid state green laser (Millennia, Spectra-Physics, Palo Alto, CA, America) as a light source, we pump the mode-locked Ti: sapphire femtosecond (fs) laser (Millennia, Spectra-Physics, Palo Alto, CA, America). The parametric conversion effect of the Ti: sapphire generates a mode-locked femtosecond pulsed laser and emits an infrared (IR) pulse laser beam with a central wavelength of 780 nm, a pulse width of 100 fs, and a repetition of 80 MHz. In the experiment, the power of the pump laser is 8.5 W, and the output power of the pulsed IR laser is about 1.4 W. We focused the IR pulsed laser through a LiB3O5 (LBO) frequency-doubling crystal to generate ultraviolet (UV) light with a central wavelength of 390 nm under the process of parametric up-conversion. In order to improve the up-conversion efficiency, a lens with a focal length of 3.5mm was inserted in front of the LBO crystal to form a small, focused beam on the crystal. Since the output beam of the femtosecond laser is elliptical, a combination of two cylindrical lenses is used to refocus the beam into a circular shape. Moreover, because the IR laser cannot be completely up-converted in the LBO crystal, the UV light beam emitted after the LBO crystal is mixed with unconverted IR light. The mixed infrared light will interfere with the fidelity of the two-photon entangled state, so we must remove it effectively. We use five dichroic mirrors (DMs) that reflect UV light and transmit IR light to form a high-efficiency filter, and the mixed IR light is effectively removed. The UV light pulse is focused by a suitable lens on a 2 mm thick β-barium borate (BBO) nonlinear crystal. By choosing a specific direction of the incident pump laser, the type II parametric down-conversion process can occur in BBO crystals. In this process, a 390 nm UV photon is split into two 780 nm IR photons with a certain probability. Due to the conservation of energy and momentum, the horizontal and vertical polarizations of the photon pair are entangled, that is, an EPR entangled photon pair. A compensator composed of a half-wave plate (HWP) and a 1 mm thickn BBO crystal behind it is used to compensate the deviation between horizontally and vertically polarized photons. A pair of entangled photons $\frac{1}{\sqrt{2}}(|H_{1}H_{2}\rangle +|V_{1}V_{2}\rangle )$ in paths 1 and 2 is prepared, where H and V represent horizontal and vertical polarization, respectively. It is experimentally found that the intensity of the UV laser strongly affects the brightness and fidelity of the EPR entangled pairs. When the average power of the UV laser pulse is reduced to about 100 mW, we can obtain better fidelity of the output state. The coincidence count rate for two-photon EPR entangled pairs is about 6 × 10^3^/s. The visibility of the EPR entangled state is about 97% on the H/V basis, and about 95% on the +/− basis, where $|+\rangle =\frac{1}{\sqrt{2}}(|H\rangle +|V\rangle )$,$|-\rangle =\frac{1}{\sqrt{2}}(|H\rangle -|V\rangle )$.

**Figure 2:**
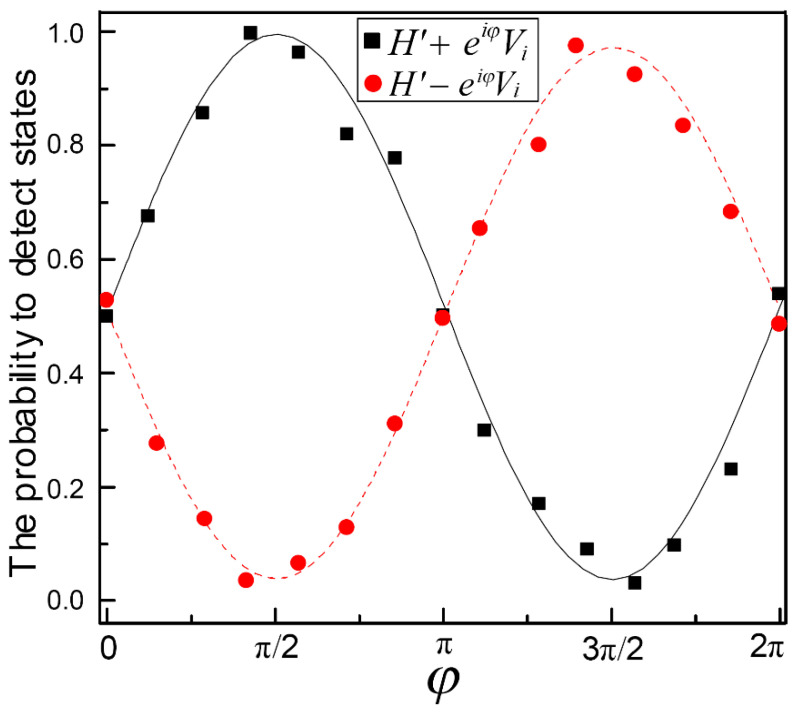
(Color online) The relationship between interference contrast and the phase of two paths.

Next, we generate the 2-D entangled state of photon polarization and spatial path. The two paths of the EPR entangled pair enter the interferometer composed of a polarizing beam splitter (PBS) and a beam splitter (BS), respectively, and obtain the 2-D four-qubit entangled state of photon polarization and a path in the 2-D entanglement generation system. In addition, the interferometer independently measures polarization and spatial qubits simultaneously on the basis of |H〉/|V〉 and $|H\rangle \pm e^{i\varphi }|V\rangle$. In the experiment, the PBS separates the photon into two possible spatial modes $H^{\prime }$ and V, according to their polarization H and V, respectively. Here, the result $H^{\prime }$ for every state analyzer represents the transmission path of the photon after the PBS before reaching the BS. The state of this single photon can now be written as an entangled state between its polarization and spatial degree. The interferometer combines the two paths of the spatial qubit to a BS. By adjusting retardation phase φ (adjusted by the thickness Δd in the optical path inserted by a quartz plate), the interference contrast between the two paths can be adjusted, shown in Figure 2, and the measurement of spatial qubits can be achieved simultaneously. The maximum contrast of the interferometer is 83 ± 0.6. Thus, 2-D entangled 4-qubit GHZ states of polarization and spatial paths can be created. The intensity of the prepared GHZ state is about 220 coincidences per second [29,30].

(1) $${|\psi \rangle }_{GHZ}=\frac{1}{\sqrt{2}}(|H^{\prime }H^{\prime }H^{\prime }H^{\prime }\rangle +|VVVV\rangle )$$

## 3. Analysis of 2-D Four-Qubit Entangled State

**Figure 3:**
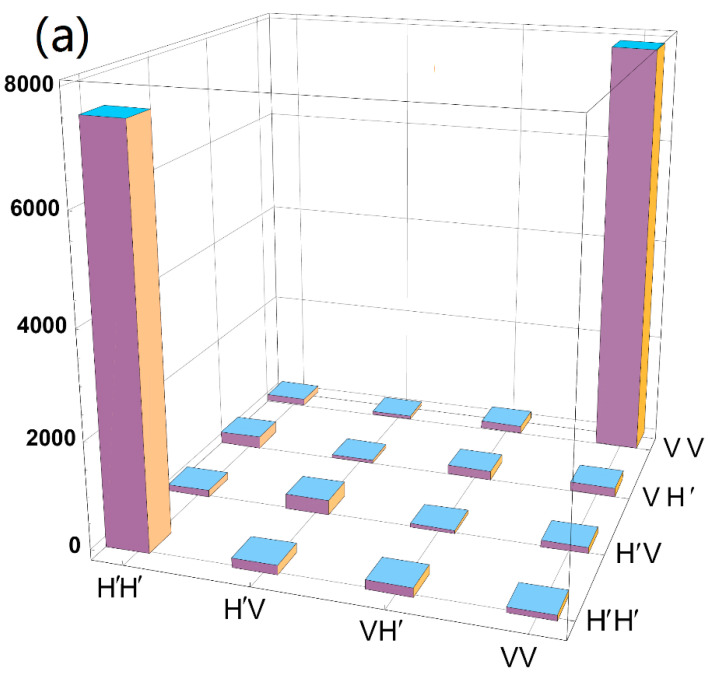

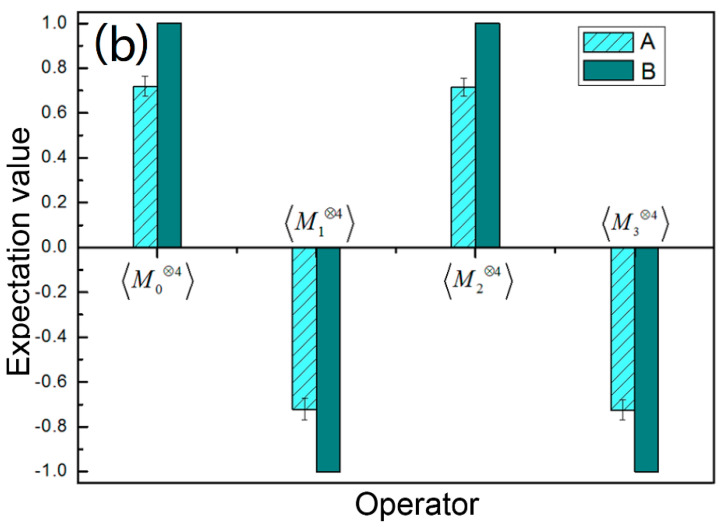
(Color online) (**a**) The density matrix of a 2-D four-qubit GHZ state on the H/V basis. (**b**) The comparison of measured and theoretical values for the entanglement witness operator, the experimental value. B, the theoretical value. Each set of coincidence measurements on the H/V, +/−, and R/L basis was 60 s. Error bar is the standard deviation derived from Poisson count statistics of raw measurement counts.

We first analyze the 2-D entangled states produced by the above steps using quantum state tomography. The prepared quantum states were analyzed using a state analyzer composed of PBS, QWP, and filters. Using the estimated density matrix in combination with full state tomography, the GHZ state fidelity can be calculated as $ℱ=Tr(\rho |\psi_{GHZ}\rangle \langle \psi_{GHZ}|)$ = 0.83 ± 0.01. Thus, with high statistical significance, a genuine four-qubit entanglement of the GHZ states created in our experiment is confirmed [31,32]. To obtain the state density matrix, a set of complementary measurements needs to be performed on the prepared GHZ states. For each state, we collect experimental data for 1296 combinations of measured underlying H/V, +/−, and R/L, where $|R\rangle =\frac{1}{\sqrt{2}}(|H\rangle +i|V\rangle )$, and $|L\rangle =\frac{1}{\sqrt{2}}(|H\rangle -i|V\rangle )$. Using these data and the maximum-likelihood technique, we reconstruct the density matrix of the 2-D entangled 4-qubit GHZ states, as shown in Figure 3. In standard error models of entangled photon measurements and counts, the counts are generally assumed to follow a Poisson distribution. Error is the standard deviation derived from Poisson count statistics of raw measurement counts. Here, we point out that the main factors affecting GHZ state fidelity include detector efficiency and double-pair effects in SPDC.

Second, to verify that ${|H^{\prime }\rangle }^{⊗2}{|H^{\prime }\rangle }^{⊗2}$ and ${|V\rangle }^{⊗2}{|V\rangle }^{⊗2}$ are indeed in a coherent superposition, we can use the following entanglement witness operator to detect the GHZ entanglement [24,25]:

(2) $$W=I/2+(|\mathrm{GHZ}\rangle \langle GHZ|{)}^{⊗4},\;$$

where ${\left(|\mathrm{GHZ}\rangle \langle GHZ|\right)}^{⊗4}$ can be decomposed into

(3) $${\left(|\mathrm{GHZ}\rangle \langle GHZ|\right)}^{⊗4}=\frac{1}{2}(|H^{\prime }\rangle {\langle H^{\prime }|}^{⊗4}+|V\rangle {\langle V|}^{⊗4})+\frac{1}{16}\sum_{0}^{3}(-1{)}^{i}{\left(M_{i}\right)}^{⊗4},\;$$

where $\langle M_{i}{}^{⊗4}\rangle =\langle {\left(\cos\theta \sigma_{x}+\sin\theta \sigma_{y}\right)}^{⊗4}\rangle$, θ=kπ/4 (k=0, 1⋯3). It can be obtained by measuring the Pauli operator of different angles. The measurement results of $M_{i}$ are shown in Figure 3b. Therefore, we can further calculate the visibility of the prepared four-qubit 2-D GHZ states at about 0.72 ± 0.015, which greatly exceeds the minimum bound of 0.5 that proves the existence of entanglement.

Furthermore, studies have shown that entangled states can unambiguously display the conflict between quantum mechanics (QM) and LR. It is known that all bi-qubit pure states violate the bipartite Bell type inequality (BTI), namely the Clauser–Horne–Shimony–Holtlemma (CHSH) inequality [33]. Studies show that the violation of inequality indicates the existence of entanglement in the system, and the amount of violation increases with the degree of the entanglement in the state [34]. We consider the Ardehali operator A in a four-qubit entangled system [35,36],

(4) $$\begin{array}{l} A=(-\sigma^{1}{}_{x}\sigma^{2}{}_{x}\sigma^{3}{}_{x}+\sigma^{1}{}_{y}\sigma^{2}{}_{y}\sigma^{3}{}_{x}+\sigma^{1}{}_{y}\sigma^{2}{}_{x}\sigma^{3}{}_{y}+\sigma^{1}{}_{x}\sigma^{2}{}_{y}\sigma^{3}{}_{y})(\sigma^{4}{}_{a}-\sigma^{4}{}_{b})\\ \;\;\;\;\;+(\sigma^{1}{}_{y}\sigma^{2}{}_{x}\sigma^{3}{}_{x}+\sigma^{1}{}_{x}\sigma^{2}{}_{y}\sigma^{3}{}_{x}+\sigma^{1}{}_{x}\sigma^{2}{}_{x}\sigma^{3}{}_{y}-\sigma^{1}{}_{y}\sigma^{2}{}_{y}\sigma^{3}{}_{y})(\sigma^{4}{}_{a}+\sigma^{4}{}_{b}) \end{array}$$

where σa=12(σx+σy), σb=12(σx−σy). *σ_x_*, *σ_y_* are the Pauli operators, which can be experimentally measured in + or − and R or L basis, respectively. For a mere classical system, that is, one which obeys a local hidden variable theory, it is well known that the correlation measure is bounded by the CHSH theorem [37]. The upper bound of operator A in the assumption of local realism is $A_{LR}=4$. This bound can be violated by an entangled quantum mechanical state, and, in fact, for any pure quantum state the correlation measure $A_{QM}$ is bounded by

(5) $$A_{QM}=\langle \Phi |A|\Phi \rangle \leq 8\sqrt{2},\;$$

which is also called Tsirelson’s bound [38]. Clearly, the QM results contradict the predictions of LR. That is to say, for a four-qubit entangled system, the expected value of operator A given by LR is not greater than 4, while the result given by the QM theory is not greater than $8\sqrt{2}$. The area between 4 and $8\sqrt{2}$ is the violation of the QM theory against the results of LR, which is not only a hot issue in quantum entanglement and nonlocality research, but also an important means to characterize the properties of entanglement resources. We verify this by performing polarization measurements on the state. The measurement of the eigenvalue of operator A is actually the joint measurement of the Pauli operator in the 2-D four-qubit system. For example, considering the joint measurement operator $\sigma^{1}{}_{x}\sigma^{2}{}_{x}\sigma^{3}{}_{x}\sigma^{4}{}_{y}$, sixteen sorts of polarization settings must be performed. For each measurement point, we collect the data of every setting $\sigma_{*}\sigma_{*}\sigma_{*}\sigma_{*}$, for 60 s and repeat it three times. After all the joint measurements are completed, we calculate that the measurement value of operator A is 8.32 ± 0.07, which shows a violation of LR with more than 61 standard deviations. This also indicates that the prepared state in the experiment is a genuine GHZ state.

## 4. Conclusions

In summary, using SPDC technology, a high brightness and high fidelity entangled two-photon state was prepared. Next, we designed a composite interferometer that can couple the polarization and spatial path of photons. Two-dimensional four-qubit GHZ states were prepared. The properties of the prepared four-qubit entangled states were analyzed by three methods. First, the density matrix of the state was reconstructed by quantum state tomography, and the fidelity was calculated to be 0.83. We also introduced an entanglement witness operator to characterize the 2-D four-qubit state entanglement. The value of the entanglement witness operator was measured to be 0.75. Further, the Ardehali inequality was shown to violate local realism by 62 standard deviations. The experimental results prove that the prepared 2-D four-photon system is an entangled state with high fidelity. There are many reasons for imperfect data. Primarily, there may be a defect in a linear optical element such as a beam splitter or filters that allows photons to be absorbed or scattered. The implementation of a high-intensity entangled source is a significant step towards practical long-distance multiparty quantum communication in the future.

H-D entanglement can be used directly in some important applications in quantum information technology. For example, it can assist us to implement many important tasks in quantum communication with one degree of freedom of photons, such as quantum dense coding with linear optics, the complete Bell state analysis for the quantum states in the polarization degree of freedom, the deterministic entanglement purification, and an efficient quantum repeater. In addition, it needs to be pointed out that it is very meaningful to further study the universality and generalization of new inequalities in multiqubit entangled states [39]. Due to the important future of quantum information technology, we all need to understand the evolution of quantum correlations under the influence of decoherence. This is also the research direction which people are quite interested in [40,41].

## Data Availability

Not applicable.
